# Implementing maternal death surveillance and response: a review of lessons from country case studies

**DOI:** 10.1186/s12884-017-1405-6

**Published:** 2017-07-17

**Authors:** Helen Smith, Charles Ameh, Natalie Roos, Matthews Mathai, Nynke van den Broek

**Affiliations:** 1 Centre for Maternal and Newborn Health, Liverpool School of Tropical Medicine, Liverpool, UK; 2 Department of Maternal, Newborn, Child and Adolescent Health, World Health Organization, Geneva, Switzerland

**Keywords:** Maternal death surveillance and response, MDSR, Implementation, Case studies

## Abstract

**Background:**

Maternal Death Surveillance and Response (MDSR) implementation is monitored globally, but not much is known about what works well, where and why in scaling up. We reviewed a series of country case studies in order to determine whether and to what extent these countries have implemented the four essential components of MDSR and identify lessons for improving implementation.

**Methods:**

A secondary analysis of ten case studies from countries at different stages of MDSR implementation, using a policy analysis framework to draw out lessons learnt and opportunities for improvement. We identify the consistent drivers of success in countries with well-established systems for MDSR, and common barriers in countries were Maternal Death Review (MDR) systems have been less successful.

**Results:**

MDR is accepted and ongoing at subnational level in many countries, but it is not adequately institutionalised and the shift from facility based MDR to continuous MDSR that informs the wider health system still needs to be made. Our secondary analysis of country experiences highlights the need for a) social and team processes at facility level, for example the existence of a ‘no shame, no blame’ culture, and the ability to reflect on practice and manage change as a team for recommendations to be acted upon, b) health system inputs including adequate funding and reliable health information systems to enable identification and analysis of cases c) national level coordination of dissemination, and monitoring implementation of recommendations at all levels and d) mandatory notification of maternal deaths (and enforcement of this) and a professional requirement to participate in MDRs.

**Conclusions:**

Case studies from countries with established MDSR systems can provide valuable guidance on ways to set up the processes and overcome some of the barriers; but the challenge, as with many health system interventions, is to find a way to provide catalytic assistance and strengthen capacity for MDSR such that this becomes embedded in the health system.

## Background

Maternal death review (MDR) has been used as an approach to improving maternal health and ending preventable maternal death in several countries. However, uptake and quality of the MDR process varies globally. While some positive experiences have been documented [1, 2], there are many countries where the MDR process ends with community or facility based reviews and there is no further follow-up action based on the findings of the review.

A new approach, Maternal Death Surveillance and Response (MDSR), which enables more robust collection and use of information for action was introduced by the World Health Organization (WHO) and partners in 2012 [3]. MDSR represents a continuous cycle of identification, notification and review of maternal deaths followed by interpretation of review findings, response and action. Whilst the maternal death review (MDR) component of MDSR is well established, “surveillance” in MDSR emphasises the need for more accurate and complete data on number of maternal deaths, and the “response” involves formulating and implementing targeted recommendations. The continuous cycle provides a means for countries to aggregate and link information on cause of and factors associated with maternal death and to examine these data to develop and implement a coordinated local and national response to prevent future deaths.

However, shifting from facility based or community based MDR to a continuous MDSR cycle that informs the wider health system requires coordination at all levels. MDSR relies on key processes including effective identification and reporting pathways, a ‘no shame, no blame’ approach to maternal death review, as well as efficient quality improvement processes at both local and national level. MDSR also requires government support with enabling policies, adequate human and financial resources, and stakeholder participation and buy-in at national and sub-national levels. A legal framework is essential to ensure that maternal death reporting is mandatory and that information generated is not used for litigation purposes. Establishing a national system for MDSR usually requires an implementation plan, and a phased approach that assumes progression towards a full-scale national MDSR system, identifying all deaths in facilities and communities, and full confidential enquiry into all deaths [3].

**Fig. 1:**
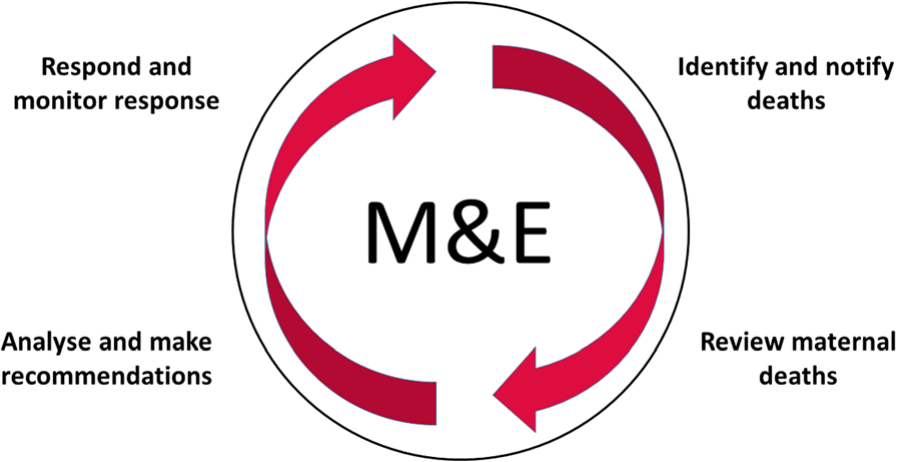
Maternal Death Surveillance and Response (MDSR) system: a continuous-action cycle (adapted from WHO 2014 [3])

The continuous cycle of MDSR has four essential components: a) identification and notifying maternal deaths, b) maternal death reviews (MDR), c) analysis and recommendations and d) response and monitoring (Fig. 1). Through its cycle of collecting, analysing, and acting upon information about maternal deaths, MDSR mirrors the steps of a typical audit or quality improvement cycle. MDR is a variant of the audit and feedback process, an established quality improvement intervention applied to many areas of healthcare. Available evidence, including a systematic review of 140 trials, suggests audit and feedback generally leads to small but important improvements in provider practice [4]. Evidence from low and middle income countries indicates that audit can improve compliance with standards and is most successful when introduced by government as a quality assurance tool with allocated resources and when audit is combined with local guideline development and targeted training [5]. Despite the emergence of more rigorous research evaluating the effects of quality improvement interventions on health outcomes, for example a cluster randomised trial in Mali and Senegal that included a focus on maternal death review [6], we still do not know enough about the overall effect of implementing fully functional MDSR cycles on maternal health outcomes. However, it is likely that just as with other quality improvement initiatives, it may work better in some contexts and settings than others [7].

WHO with UNFPA recently initiated the Global MDSR Implementation Survey [8] among member states which aims to provide information on the degree of implementation and allow tracking of progress over time. The first survey was completed in 2015 and included country progress data and case studies describing the MDSR implementation. Eighteen countries responded to the survey’s request for case studies, which provide examples of how MDSR policy is converted to practice, how MDSR elements are implemented in different contexts, and barriers to full functioning of the MDSR cycle [8]. In this paper, we highlight factors related to the successful implementation of maternal death review and response, and draw out key challenges in countries where implementation has been less successful. The 10 countries included in this analysis implement different types of maternal death review: those with an established system for MDSR implement national and sub-national confidential enquiry into maternal deaths at facility and community level (UK, RSA, Malaysia); those where MDR implementation is ongoing utilise facility based maternal death review (Kenya), and national (Moldova) and sub-national (India) confidential enquiry; and those where MDR has recently been introduced are using facility based maternal death review (Nigeria, Cameroon, Malawi) and verbal autopsy (Bangladesh). All approaches have in common the purpose of collecting information on maternal deaths in order to learn about causes of death and identify remedial actions to prevent further deaths. These are not uncomplicated processes to establish; all have important prerequisites and need to be adapted to country resources and requirements.

In this review, we analyse 10 country case studies in order to determine whether and to what extent these countries have implemented the four essential components of MDSR and identify lessons for improving implementation.

## Methods

We conducted a secondary analysis of 10 case studies, selected from those submitted to the WHO Global MDSR Implementation Survey to represent countries at different stages of MDSR implementation: Bangladesh, Cameroon, Malawi, and Nigeria (where MDR is being introduced); India, Kenya and Moldova (implementation of MDR is ongoing); and Malaysia, South Africa, and UK (successful implementation of MDR, surveillance and response at national level). To produce this secondary analysis we also draw on a series of articles on experiences of implementing maternal death review published in a special supplement on international reviews: quality of care in BJOG: an International Journal of Obstetrics & Gynecology [9]. Case studies are in-depth descriptions of naturally occurring cases [10], are a method for studying planned change in real world settings, and particularly valuable in understanding why interventions succeed or fail. Each case study included in this paper describes a country experience of implementing notification and review of maternal deaths, and identifies achievements and challenges. We conducted a secondary analysis of these cases to explore and compare country experiences collectively. Taken as a whole, the case studies offer important insight into the factors that can lead to successful transition from MDR to MDSR.

We applied a policy analysis framework [11] to draw out lessons learnt and opportunities for improving implementation in countries moving from a policy for maternal death review to a continuous MDSR cycle. The original policy analysis model (or policy ‘triangle’) proposed by Walt in 1994 identified four highly interrelated dimensions of a health policy that can affect its development and implementation (actors, context, process and content) [11, 12]. The model supposes that ‘actors’ responsible for developing and implementing a policy can be influenced by the context in which they work; in turn ‘context’ (societal or macro level as well as the health sector context or micro level) is affected by changes in political, economic and social factors. The policy ‘process’, including the strategies for implementation at national and sub-national level, is affected by actors and their values, intentions and expectations; and the ‘content’ of a policy will be the result of any or all of the other dimensions. The model has been applied in analyses of various health policies including factors important in the scale-up of maternal and newborn health interventions [13], the development of oral health policy in Nigeria [14] and strengths and weaknesses of policy processes in maternal health in Vietnam, India and China [15]. In line with recent applications of the framework [16], we conducted a comprehensive analysis exploring the independent and mutual influences of context, actors and process on the development and implementation maternal death review policies in our case study countries. One author (HS) systematically extracted information about context, actors, process, and implementation lessons from the available case study and published article for each country. Three authors (HS, CA, NvdB) examined these components across the 10 case studies and specifically in relation to the different stages of implementation. Through discussion we identified the consistent drivers of success in countries with well-established systems for MDSR, and common barriers in countries where the transition from MDR to MDSR is yet to take place.

## Results

In this section, we describe how countries at different stages of MDR implementation have developed and implemented policies on MDR, using context, actors and process as an organising framework (Table 1). We draw on the case studies produced for the Global MDSR Implementation Survey, and returned to the original publications where needed, to provide illustrative examples.

**Table 1:** Characteristics of country case studies on MDSR

Source article	Country	Case study title	Political context	Key actors	Type of maternal death review and scale of coverage
Countries with an established national level MDSR system					
Ravichandran 2014 [19]	Malaysia	Lessons from the confidential enquiry into maternal deaths, Malaysia	Government scaled up the existing system of maternal mortality audit and introduced the National Confidential Enquiry into Maternal Deaths (CEMD) in 1991.	Director-General of Health State Director of Health and State Obstetricians The MoH absorbs the cost of the CEMD and the Family Health Division (FHD) acts as Secretariat.	National confidential enquiry All deaths in facilities and communities
Moodley 2014 [17]	South Africa	The confidential enquiry into maternal deaths in South Africa: a case study	Free health care for pregnant women and children (1994). Maternal deaths became notifiable by law (1997). National Committee for Confidential Enquiry into Maternal Deaths (NCCEMD) established (1998).	NCCEMD is a ministerial committee, with representatives from obstetrics, gynaecology and midwifery cross South Africa’s nine provinces. The Department of Health provides financial and administrative support to the NCCEMD.	National confidential enquiry Deaths in all facilities
Kurinczuk 2014 [16]	UK	Experiences with maternal and perinatal death reviews in the UK - the MBRRACE - UK programme	Original CEMD established (1954) Confidential Enquiries into Maternal and Child Health (CEMACH) (2003) Maternal Newborn and Infant Clinical Outcome Review Programme (MNI-CORP) (2012)	MBRRACE-UK is a collaboration which assesses the process. Led by the National Perinatal Epidemiology Unit at University of Oxford (members from the Universities of Leicester, Liverpool and Birmingham; University College London and the Stillbirth and Neonatal Death charity (SANDS)). MBRRACE is commissioned by the Healthcare Quality Improvement Partnership (HQIP) to oversee MNI-CORP.	National confidential enquiry All deaths in facilities and communities
Countries where MDR is ongoing					
Paily 2014 [18]	India	Confidential review of maternal deaths in Kerala: a country case study	Facility-based maternal death audit initiated by the Director of Health Services (2000) Kerala Federation of Obstetrics and Gynaecology (KFOG) assumed leadership for maternal death review (2002). KFOG implemented a Confidential Review of Maternal Deaths (CRMD) based upon the UK system of CEMD (2004).	KFOG provides the central secretariat. The Department of Health (DoH) of the Government of Kerala supports the programme.	State level confidential enquiry Facility deaths only
Ameh 2015 [12]	Kenya	DFID programme experience implementing MDSR	Government of Kenya made maternal death notification mandatory (2004). Maternal death review (MDR) system established (2004). Free maternity services were introduced in Kenya (2013).	Support from the Centre for Maternal and Newborn Health (CMNH) at Liverpool School of Tropical Medicine (LSTM) Kenya Ministry of Health (MoH)	Facility-based National coverage
Hodorogea 2014 [20]	Moldova	The Moldovan experience of maternal death reviews	Recognising the deficiencies in the death review system, the MoH implemented a new model similar to the UK (2003).	Support from World Health Organization and UNICEF Ministry of Health (MoH) National CEMD Committee.	National confidential enquiry All deaths in facilities and communities
Countries where MDR is being introduced					
Halim 2014 [15]	Bangladesh	Cause of and contributing factors to maternal deaths; a cross-sectional study using verbal autopsy in four districts in Bangladesh	Verbal autopsy (VA) part of the Demographic and Health Survey in Bangladesh (1990) Introduced across four districts as a method to be used to review all maternal deaths in these districts (2010)	Government of Bangladesh The Directorate General of Health Services Centre for Injury Prevention, Health Development and Research UNICEF Bangladesh provided funding through a Joint UN-Government project.	Verbal autopsy Sample of districts
de Brouwere 2014 [13]	Cameroon	Achievements and lessons learnt from facility-based maternal death reviews in Cameroon	Cameroon adopted the Campaign on the Accelerated Reduction of Maternal Mortality in Africa (CARMMA) as its guiding strategy (2010). This included the introduction of maternal death review (MDR) at facility and community levels.	Society of Gynaecologists and Obstetricians of Cameroon (SOGOC) via the International Federation of Gynaecology and Obstetrics - Leadership in Obstetrics and Gynaecology for Impact and Change (FIGO-LOGIC) project Ministry of Public Health	Facility-based Urban hospitals
Owolabi 2014 [14]	Malawi	Establishing cause of maternal death in Malawi via facility-based review and application of the ICD-MM classification	WHO developed a standard method for classifying maternal and pregnancy-related deaths. Quality improvement programme at four referral hospitals and four health centres in one district applied the ICD-MM in facility-based maternal death reviews (2011).	Collaboration between the Centre for Maternal and Newborn Health (CMNH) at Liverpool School of Tropical Medicine (LSTM), the Ministry of Health MoH Malawi and UNICEF Malawi	Facility-based Sample of districts
Achem 2014 [11]	Nigeria	Setting up facility-based maternal deaths reviews in Nigeria	Government of Nigeria has increased funding and instated policies and programmes directed at improving maternal health. After a previously unsuccessful attempt (2003), MDR was approved as part of the national strategy for improving maternal health care (2013).	Society of Gynaecology and Obstetrics of Nigeria (SOGON) via the International Federation of Gynaecology and Obstetrics - Leadership in Obstetrics and Gynaecology for Impact and Change (FIGO-LOGIC) project Technical assistance from the UK and South Africa National Council on Health	Facility-based National coverage

### Context factors influencing the introduction of MDR and response

#### Strong government commitment

In countries with well-established confidential enquiry into maternal deaths (CEMD), national committees were often set up following on from increased government commitment evident in policies and laws directed at improving maternal health. For example, in the Republic of South Africa (RSA) the policy on free healthcare for women and children in 1994 and the law on notification of maternal deaths in 1997 were precursors to the national committee for Confidential Enquiry into Maternal Deaths in 1998 [17]. In Malaysia, a long history of political commitment to strengthening maternal and child health services culminated in several specific initiatives including the scale up of the existing system of maternal mortality audit to a formal national level process [18].

In countries with an established MDSR system, government commitment and involvement is also evident through financial support for review activities, and administrative support through hosting secretariats and coordinating bodies within government institutions (Malaysia and RSA). The UK has the longest running system for maternal death review and the methodology used is considered the global standard. Sustained political commitment and rounds of restructuring have allowed the system to evolve to include a review of serious maternal morbidities alongside the decreasing number of cases of maternal mortality [19].

In countries where implementation is ongoing or being introduced, MDR was often introduced by governments within a context of global commitment to the elimination of maternal deaths, broad national political support directed at improving maternal health. For example, in Nigeria the government increased funding, instigated policies for improving maternal health and approved MDR as part of the national strategy [20], and in Kenya the government made maternal death notification mandatory, introduced a policy of free maternity services, and established MDR as routine [21]. In some cases, international and regional advocacy campaigns helped drive country efforts to take action, such as the adoption by Cameroon of the Campaign for Accelerated Reduction of Maternal Mortality in Africa (CARMMA) as its guiding strategy [22] and the influence of the Commission on Information and Accountability (CoIA) in Kenya [21]. In Bangladesh, verbal autopsy was originally part of the Demographic and Health Survey (DHS), but 20 years later the government introduced it into the existing health system structure [23].

### Professional organisations as drivers of MDR

A common feature in well-established systems of the UK and RSA CEMD is ownership by the professional OBGYN association. Members serve as assessors and on the relevant committees free of charge. This is critical for sustainability as low resource countries progress from facility based reviews to more in-depth regional or national CEMDs. In countries where MDR implementation is ongoing there is also evidence of the critical role of professional associations. For example, in India the Kerala Federation of Obstetrics and Gynaecology (KFOG) assumed leadership for maternal death review, instigated state level confidential enquiry and provides the central secretariat for maternal death review [24].

### Adequate legal frameworks

Health workers are less likely to report maternal deaths and provide information about those deaths if they fear punitive action, so another key factor is adequate legal frameworks. There is little information on such frameworks or how they are maintained, but our case studies highlighted ways in which countries had enabled a confidential and non-punitive system. In RSA, where national level MDR is well established all information received by the committee is completely anonymised and destroyed once reports are published, and importantly relevant judicial bodies have ratified the process so that data collected and review forms used by the CEMD process cannot be used in litigation or disciplinary processes [17, 25].

### A ‘no shame, no blame’ culture

In Malaysia MDR is conducted as a ‘no shame, no blame’ process, with clear emphasis on learning from each death to improve the system. The term ‘substandard care’ originally used in the Malaysian MDR system to categorise inappropriate or deficient care was changed to ‘remediable factors’ to create a more positive image of the care provided and the caregivers; it also helped to emphasise that many factors contributing to a maternal death are beyond the control of an individual [18]. Countries at different stages of MDSR implementation highlighted a need to reassure health professionals involved in MDR, and take action to avert or overcome a blame culture [21, 24, 26]. In Kenya, there is recognition that without an adequate legal framework and sensitisation of health workers to the ‘blame free’ principle government plans to progress the MDR system may stall [21].

### Key actors involved in driving implementation of MDR

In most case studies, national Ministries and Departments of Health have been key actors in implementing policies to facilitate MDR at national and subnational level. WHO supported these policy level changes and, in partnership with UNFPA, UNICEF, national professional societies and others, guided the implementation of MDR in many countries. National and sub-national obstetric and gynaecological societies, supported by the International Federation of Obstetrics and Gynecology (FIGO), played key roles. In many cases these societies were wholly responsible for initiating implementation of MDR through for example: advocacy and organising training workshops in Nigeria [20]; monitoring MDR reporting in Cameroon [22]; and providing the central secretariat for confidential enquiry in Kerala State, India [24]. Most case studies acknowledge the support of WHO and other technical experts in setting up national systems for MDR. For example, in Nigeria experts from the UK and RSA provided technical assistance, and the Centre for Maternal and Newborn Health at Liverpool School of Tropical Medicine has helped strengthen the national MDR system in Kenya [21] and facility-based MDR in Malawi [27]. UNICEF supported the MDR activities in Bangladesh [23], Malawi [27], and Moldova [26]. In RSA and Malaysia, strong national government commitment has been evident from the early stages, through financial support to review activities at national level [17, 18]. In Malaysia, RSA and the UK the government provides administrative support via secretariats and national committees. In Malaysia State Directors of Health are even present at death review meetings. In the UK, although mandated, supported and funded by the Department of Health, the conduct of confidential enquiries is led by a consortium of academic institutions [19].

The case studies of countries with established national level MDR systems highlight the commitment of doctors, midwives and other personnel who participate, without extra pay, in the intensive process of assessing cause of death, preventable conditions and contributing factors. In well-functioning systems, the support of professional organisations or colleges is important in facilitating this. In the UK, all doctors are required to participate in audit as part of professional development. Similarly, in RSA independent provincial level assessors include obstetricians, medical officers, midwives and anaesthetists who are not remunerated as audit is considered a professional duty. In Malaysia, it is considered an honour and mark of esteem to be invited to be on the CEMD committee.

### Identification and notification of maternal deaths

A national level MDSR system requires accurate information on all deaths that occur in women of reproductive age in facilities and in the community and this requires a level of sophistication in vital registration. In countries with an established MDSR system, for example the UK and Malaysia, routine notification of deaths from facilities alongside the formal vital registration system (including indicating maternal deaths on death certificates of women) enables efficient identification of all maternal deaths [18, 19]; in RSA most deaths reported are from facilities with no system currently for routinely identifying deaths in the community, assuming a certain degree of underreporting [17]. However, in all three countries more than 90% of births are attended by skilled health staff, which allows for facility-based identification, notification and review of maternal deaths. In other countries where MDR is being introduced or is not yet fully national scale, notification is via a combination of lead individuals at facilities (India, Kenya, Malawi), district level coordinators who rely on reports from colleagues, the media or hearsay (India), and in some cases, legal requirement to enforce maternal death notification (Kenya, Nigeria). In countries where vital registration systems are weak, skilled birth attendance rate is low and many deaths occur without any contact with formal health facilities, identification and notification of maternal deaths at community level is a critical source of information. But it is more difficult to establish community based MDR and key challenges include inaccurate reporting and classification of deaths based on information provided by community members, and lack of effective and regular supervision of the process [23].

### Maternal death review

Reviewing the maternal deaths that occurred in facilities and communities relies on a degree of data collection at facility level, and verification of that data, combined with information received from community sources. Review of maternal deaths in a fully functional MDSR system typically has a proper structure to coordinate MDR. For example, in Malaysia MDR committees sit at state level and are overseen by a national secretariat [18]; in RSA, provincial level coordinators receive data from facilities, coordinate assessment of deaths by provincial assessors, and liaise with the national MDR committee for quality assurance and reporting [17]; and in the UK, a collaboration of academic institutions coordinate the confidential enquiry process supported by consultant assessors appointed by the Royal Colleges [19].

Many of the case studies of countries where MDR is being introduced or implementation is ongoing revealed a lack of knowledge among facility staff of the reporting process, which led to poor compliance, incomplete and inaccurate reporting, and failure to dispatch case records to central committees [20, 24, 27]. Poor flow of information from facility to district or central committees is a common challenge and countries wishing to scale up will need to develop an enabling coordination structure and promote collaboration between different levels participating in review. However, even in well-established systems like the UK CEMD, the number and quality of local facility reports is sometimes lacking and the central review committee rarely receives all reports of maternal deaths [28]. Whereas electronic data collection and reporting systems reduce delays and improve data accuracy, they may potentially lack detail. Hand written notes may suffer from lack of legibility but do often contain sufficient information in the narrative. Inclusion of an external assessor or consultant in the MDR committee meeting seems to improve the objectivity and quality of the reviews.

Where MDR is being introduced or is ongoing, a culture of improvement has often not been sufficiently developed for staff to perceive a benefit of MDR [22, 24, 26]. The concepts of evidence-based practice and improvement through comparison of case management against national standards may not be familiar to all cadres involved and this can impede the process of review, and ultimately response and action to rectify identified problems with care. Strong leadership, dedicated MDR teams and coordinators, training and regular coaching and supervision may help to improve understanding of the rationale for continuous improvement [22]. Alongside this there is need for staff involved in MDR to use clear criteria and a standard classification system to evaluate cause of death. In 2012 WHO developed a new classification system, the International Classification of Diseases-Maternal Mortality (ICD-MM), for consistent and accurate identification and classification of the causes of maternal deaths. The new ICD-MM classification has been piloted using existing data from five countries [29], and pilot tested in one district in Malawi [27] although it is not yet applied universally in the countries we analysed.

A challenge in all countries is identifying sustainable resources (financial and human) for MDR. Programmes to establish MDR have tended to be funded initially by development donors or partners, for example the International Federation of Gynecology and Obstetrics- Leadership in Obstetrics and Gynecology for Impact and Change (FIGO-LOGIC) project in Cameroon, Kenya, Malawi and Nigeria; once the project ended all training ceased and the government has not allocated funds to scale up [20–22] In these countries professional societies have also taken on much of the coordination and implementation of MDR; without the goodwill of small teams of committed individuals MDR would not happen [17, 24]. Without government commitment and funds to scale-up countries are unable to continue to strengthening capacity of staff at all levels to conduct MDR – i.e. training on the MDR method in all facilities, and training for assessors on completing MDR forms, maternal death classification (using ICD-MM) and formulating recommendations [22, 27, 29]. In Nigeria, the government has proposed plans for the scale up of the MDR programme, but poor budget allocation for health and low prioritisation and poor planning for MDSR are significant barriers to making this happen [20]. Setting up community based maternal death review also requires time, effort and resources, particularly since it depends on training community health workers to routinely identify maternal deaths and complete death notification forms (usually with little supervision), collection of information surrounding the death via household interview, and a process to ensure this information is fed upwards to district MDR committees [23].

### Analysis and recommendations

Countries with well-established MDR systems make use of technology to support data analysis, which makes for more rapid aggregation and analysis of information from across districts or states. For example, in the UK a secure web-based system improves case note viewing and assessment, while in RSA a specially developed electronic database Maternal Morbidity and Mortality Assessment System (MaMMAS) saves time in collating and reporting maternal death data from across nine provinces. Other countries scaling up MDR have begun to adapt and adopt the MaMMAS system (e.g Malawi, Kenya) with technical support from the software developer in RSA. Ultimately this allows for systematic identification of actions and more efficient reporting but requires constant monitoring of data entry and outputs. Few of the case studies of countries where MDR is being introduced mentioned processes for data analysis, and poor flow of data from district to national level can affect the quality and quantity of information available for analysis and recommendations [27].

### Response and monitoring

Countries with well-established national systems for MDR have systematic processes in place for identifying remedial actions, publishing and disseminating reports and strong links to relevant ministries or departments to ensure prompt dissemination and implementation of national committee recommendations [17–19]. In the UK, CEMD reports provide recommendations for action at national, district and facility level, and include vignettes of stories generated through confidential enquiry which can act as powerful arguments for changing practice [19]. In Malaysia CEMD reports provide information directly to government to support budget requests to target services that needs strengthening, and service level key performance indicators integrate factors from the CEMD to ensure improvements are sustained [18]. However, CEMD reports are usually published triennially, and in high burden countries, it may not be feasible to wait this long to make changes. Malaysia is currently trialling a hybrid approach of initiating actions annually at national level and every 3 to 6 monthly at subnational level which might be more applicable in countries introducing MDSR [30]. Despite having systems to rapidly identify response actions, and wide dissemination, these countries still struggle to implement actions.

Countries with less well established MDR systems also find it difficult to implement review recommendations; country case studies from Kenya and Cameroon cite lack of planning for implementation and monitoring of responses and challenges in the health system as impediments to action [21, 22]. Successful implementation often depends on the ability of the health system to respond, especially since some problems cannot be solved immediately at facility level. Countries recognise the need for a formal government supported mechanism or plan to support continual improvements in quality of care at all levels [21, 22, 24]. Moldova, a country taking first steps to national CEMD, plans to officially launch future CEMD reports with media, local authority and women’s organisations present, to overcome challenges of reaching all key stakeholders with the findings [26].

For the MDSR cycle to work efficiently as a continuous quality improvement process to prevent future maternal deaths, certain inputs and processes need to be in place at facility, district and national level. Table 2 summarises the drivers and conditions of success in countries with an established MDSR system, and aspects that need strengthening in countries transitioning from MDR to MDSR. This review of country experiences highlights the need for a) social and team processes at facility level, for example the existence of a ‘no shame, no blame’ culture, and the ability to reflect on practice and manage change as a team for recommendations to be acted upon, b) health system inputs including adequate funding and reliable health information systems to enable identification and analysis of cases c) national level coordination of dissemination, and monitoring implementation of recommendations at all levels and d) mandatory notification of maternal deaths (and enforcement of this) and a professional requirement to participate in MDRs.

**Table 2:** Key drivers of success and aspects that need strengthening to implement MDSR

Drivers and conditions of success	Relevant case study examples	Aspects of implementation that need strengthening as countries transition from MDR to MDSR	Relevant case study examples
Policy level		Policy level	
Strong government commitment and involvement in commissioning or providing administrative support to the CEMD process	Malaysia, RSA, UK	Less reliance on external funds and/or the goodwill of national professional organisations to support administration, training and implementation of the MDR process	Cameroon, India, Kenya, Malawi, Nigeria
Enforcement of MDR policies by professional organisations/colleges	UK	Political commitment and government funds to scale-up, supervise and monitor MDR activities	Bangladesh, Cameroon, India, Nigeria
Adequate legal frameworks to prevent punitive action	UK, Malaysia		
Use of review data to target MoH budget allocation and revise key performance indicators	Malaysia		
District level		District level	
Accurate data on number of live births and maternal deaths collected via reliable district health information systems or routine death registration	Malaysia, RSA, UK	Knowledge among health professionals and administrators of the MDSR reporting process	India, Nigeria, Malawi
Electronic systems that allow for rapid assessment and analysis	Malawi, RSA, UK	Available reporting forms or forms to collect information pertaining to maternal deaths that are fit for purpose	Kenya, Malawi
Systematic identification and dissemination of remedial actions and recommendations targeted at different levels of the health system	Malaysia, UK	Strategy for monitoring implementation of recommendations	Cameroon, Kenya
		Obtaining accurate patient records or information on circumstance and management of women at all levels	Bangladesh, India, Malawi, Moldova, RSA
		Underreporting and misclassification of maternal deaths	Bangladesh, India, Kenya, RSA
Facility level		Facility level	
Commitment of unpaid health professionals who participate as part of professional development	Malaysia, RSA, UK	Familiarity and confidence in the reporting process for MDR	India, Kenya, Nigeria
		Knowledge and understanding among healthcare providers of how to assign cause of death and contributing factors and/or apply ICD-MM	Kenya, Malawi
		Need to reassure health professionals involved in MDR of the principles of confidentiality and anonymity, and take action to avert or overcome a blame culture	India, Kenya, Malaysia, Moldova, RSA
		Culture among assessors and/or healthcare workers of quality improvement through reflection on practice	Cameroon, India, Moldova, Nigeria
		Mechanism to support health facilities or health professionals to act on review recommendations to improve quality of care at different levels	Cameroon, India, Kenya. Moldova, RSA

## Discussion and conclusion

This review set out to describe how countries have developed and implemented MDSR, summarise factors related to the successful implementation of a fully functional national level system, and analyse key challenges in countries where MDR is just beginning or is ongoing. Overall, although MDR is at least introduced, accepted and ongoing at subnational level in many countries, it is not institutionalised at national and subnational level and the shift from facility based MDR to continuous MDSR that informs the wider health system still needs to be made.

### Study limitations

Our analysis draws on a small number of country case studies only. However, there is little published information on MDSR implementation experience so we actively sought this and included countries at different stages of transition from MDR to MDSR. We appreciate that there may be additional lessons to be learnt from other countries, and that not all of the lessons we highlight will be applicable in all countries. Case studies provide in-depth descriptions of intentional processes of change such as MDSR, and the information for the case studies was generated by individuals closely involved in the process. This analysis is a starting point for identifying the pitfalls to and prerequisites to scaling up MDSR to national level and ensuring continuous surveillance and response. What is now needed is a more objective and comprehensive assessment of what is working where and why to understand how MDSR can be implemented optimally. This will complement WHO efforts to track progress through the global implementation survey, and allow for assimilation of lessons learnt with future plans for surveillance and response systems for stillbirths and perinatal deaths.

Implementing actions in response to recommendations appears to be a weak point in the MDSR system, which even countries with well-established national processes struggle with. In many ways this mirrors the situation in other types of audit used in maternal and newborn health. A problem with the audit or review process on which MDSR is predicated is the assumption that once quality of care problems are identified and made known to health professionals, policy makers and others there will be a self-correction in behaviour. In reality, implementation of identified solutions or recommendations may be the hardest part of the continuous improvement cycle. The way that audit or review data is used, and specifically how feedback is designed and delivered, can influence the likelihood that change will occur. At facility level, timely, individualised and non-punitive feedback following audit is more likely to lead to ‘action’ [31].

Part of the challenge is that the MDSR cycle operates on a much larger scale than facility review, and the ‘response’ component is all-encompassing - involving all levels of the health system, multiple evidence-based interventions, and engagement of multiple stakeholders, at different time points. This wide ranging and often shifting process can be overwhelming and none of our case studies elaborated on how response implementation happens or who is responsible for facilitating and monitoring responses at each level. We think this is an area that deserves further investigation to find out what works and what does not, and how to improve this critical component of MDSR.

WHO recommends a phased approach to MDSR implementation, which reflects progression along several dimensions [3]. In Malaysia, RSA, and the UK the system has evolved over time into an efficient mechanism for reporting and review of maternal deaths with formulation of focused and strategic prioritised recommendations for action, and the developmental steps are evident. For example, in Malaysia the government has supported a shift from subnational facility-based maternal death review to full national level confidential enquiry of all deaths, and key performance indicators in public service have evolved to integrate major factors from the CEMD so that improvement is sustained [18]. RSA has an established national system for CEMD but lacks a process for capturing deaths in communities; but with further development of district health information systems this will be overcome [17]. In Bangladesh, there is recognition of the drawbacks of only reviewing maternal deaths in the community and a desire to complement this with facility based review [23]. There is evidence of evolution in the UK system to incorporate near miss and perinatal mortality audit [28]. As with other health systems interventions there is an argument that one cannot wait for the system to evolve; in the case of MDSR it requires inputs at all levels of the health systems and if these are set up effectively they can complement and strengthen existing routine health surveillance and reporting processes.

Countries with established MDSR systems can advise on ways to set up the processes and overcome some of the barriers; but the challenge, as with many health system interventions, is to find a way to provide catalytic assistance and strengthen capacity for MDSR such that this becomes embedded in the health system.

## Abbreviations

CARMMA: Campaign for Accelerated Reduction of Maternal Mortality in Africa
CEMD: Confidential Enquiry into Maternal Deaths
CoIA: Commission on Information and Accountability
FIGO: International Federation of Obstetrics and Gynecology
FIGO-LOGIC: International Federation of Gynecology and Obstetrics- Leadership in Obstetrics and Gynecology for Impact and Change
ICD-MM: International Classification of Diseases-Maternal Mortality
KFOG: Kerala Federation of Obstetrics and Gynaecology
MaMMAS: Maternal Morbidity and Mortality Assessment System
MDR: Maternal Death Review
MDSR: Maternal Death Surveillance and Response
RSA: Republic of South Africa
UNFPA: United Nations Population Fund
UNICEF: United Nations Children’s Fund
WHO: World Health Organization
